# Feasibility and Preliminary Efficacy of an m-Health Intervention Targeting Physical Activity, Diet, and Sleep Quality in Shift-Workers

**DOI:** 10.3390/ijerph16203810

**Published:** 2019-10-10

**Authors:** Stina Oftedal, Tracy Burrows, Sasha Fenton, Beatrice Murawski, Anna B. Rayward, Mitch J. Duncan

**Affiliations:** 1 School of Medicine & Public Health, Faculty of Health and Medicine, The University of Newcastle, University Drive, Callaghan, NSW 2308, Australia; sasha.fenton@uon.edu.au (S.F.); beatrice.murawski@newcastle.edu.au (B.M.); anna.rayward@uon.edu.au (A.B.R.); mitch.duncan@newcastle.edu.au (M.J.D.); 2 Priority Research Centre for Physical Activity and Nutrition, The University of Newcastle, University Drive, Callaghan, NSW 2308, Australia; tracy.burrows@newcastle.edu.au; 3 School of Health Sciences, Faculty of Health and Medicine, The University of Newcastle, University Drive, Callaghan, NSW 2308, Australia

**Keywords:** multiple lifestyle behaviors, health promotion, nutrition, behavior change, resistance training, exercise

## Abstract

Poor health behaviors are prevalent in shift-workers, but few multiple health-behavior interventions consider their unique needs. This study aimed to (1) evaluate the feasibility and acceptability of an existing app-based intervention to improve physical activity, diet, and sleep quality in a shift-worker population, (2) estimate intervention effect in a four-week pilot randomized controlled trial (RCT) (ACTRN12618001785291). Shift-workers (18–65 years old) were randomized to intervention (*n* = 20) or wait-list (*n* = 20) groups. Outcomes included recruitment, engagement, attrition, usefulness ratings, System Usability Scale (SUS), qualitative interviews, and estimation of treatment effect (minutes of physical activity, diet quality, and sleep quality) using mixed model analysis. Recruitment took one week. App-use at week four was 55% (11/20), 85% (34/40) completed the four-week follow-up questionnaire, and 20% (4/20) of the intervention group completed the qualitative interview. The intervention was rated as slightly to moderately useful by 76.9% (10/13) of participants on a five-point scale. The SUS score was 62.7 (12.7) out of 100. Diet quality improved for the intervention (4.5 points; 95% confidence interval (CI) = 0.1, 8.9; *p* = 0.047) vs. the wait-list group, but not physical activity or sleep quality. Qualitative interviews found that a more tailored intervention, more accessible information, and interactive features were desired. The intervention was feasible in terms of recruitment, but modifications to increase engagement are needed.

## 1. Introduction

Around 16–20% of the workforce in Europe and Australia is represented by shift-workers who have work hours outside of the standard daytime work hours (7:00 a.m. to 6:00 p.m.), including both evening and late-night work [1,2]. They play a vital role in the 24/7, 365-day operations of many industries, yet this comes at the cost of an increased risk of all-cause mortality, cardiovascular disease (CVD), type 2 diabetes, metabolic syndrome, and poor mental health for shift-workers [3,4,5,6]. The risk of any CVD event is 17% higher among shift-workers than non-shift-workers, and the risk of CVD and coronary heart disease (CHD) mortality is 20% higher [7].

The poorer health status of shift-workers is in part driven by external factors (e.g., work hours) that alter their circadian rhythm, which in turn influences their metabolism and other physiological processes, including increased stress and allostatic load [5,8]. Shift-workers’ poorer health is also influenced by more modifiable factors such as engaging in high-risk lifestyle behaviors [9]. The unique demands of shift-work include the need to resist the homeostatic drive to sleep at night and live on a schedule that is not in sync with that of their family or society at large, and that is frequently changing (e.g., rotating shifts, on/off roster). Consequently, the ability to prioritize, schedule, and allocate enough time for health-promoting lifestyle behaviors such as sufficient physical activity, eating a good-quality diet, and getting enough good-quality sleep [10].

Poor sleep health is the most commonly reported high-risk behavior in shift-workers [11,12] and significantly increases the risk of chronic disease [13,14]. Circadian misalignment occurs when our behavioral cycles like sleep/wake and fasting/feeding are mismatched with the endogenous circadian rhythms. This can result in dysregulation of feeding behaviors, as well as changes in appetite-stimulating hormones, glucose metabolism, blood pressure, temperature, and heart rate [15]. Differences in dietary patterns between shift- and non-shift-workers have been reported [16,17,18]. Specifically, greater frequency in eating events, high-fat snacks, and caloric intake peak during the night shift was demonstrated in shift-workers, as well as a shorter maximum fasting period, and this could generate unfavorable metabolic responses such as hyperlipidemia and hyperglycemia, both increasing chronic disease risk [18,19,20,21,22]. Reports on differences in physical activity between shift- and non-shift-workers are mixed [23], and may have a stronger association with occupational tasks rather than shift pattern [24]. However, a recent study of female nurses found that both rotating shift work and unhealthy lifestyle, as assessed by low physical activity level, poor diet quality, body mass index over 25 kg/m^2^, and current smoking, were associated with a higher risk of type 2 diabetes, and the joint effect of these four risk factors was higher than the addition of the risk associated with each individual factor [6]. This indicates that shift-workers may benefit from a multiple health-behavior change over and above that of non-shift-workers [6].

Although many lifestyle behavior interventions exist, relatively few specifically target shift-workers, and those that do utilize resource-intensive methods such as one-on-one consultations and financial incentives which make scaling the program to reach a large number of shift-workers unfeasible [25,26,27]. A useful approach may be to modify existing efficacious lifestyle interventions, but little evidence exists on how to modify “typical” lifestyle behavior interventions in order to suit the unique requirements of shift-workers [28]. Utilizing mobile health (m-health) may be especially suitable in this population due to the ease of access any time of the day or night, the low cost involved with scaling the intervention, and the lack of geographical barriers. As few multiple behavior interventions exist for shift-workers, we conducted a feasibility study of an existing m-health intervention with process evaluation to inform the co-design of m-health intervention tailored to the unique needs of shift-workers. The current study aims were as follows:

1. To evaluate the feasibility of an intervention aimed at improving physical activity, diet, and sleep quality not tailored to shift-workers (Move, Eat, and Sleep [29]) in a shift-worker population and receive feedback on improvements to increase acceptability.
2. To estimate the effect of the shift-worker Move, Eat, and Sleep intervention on improving physical activity, diet quality, and sleep quality in shift-workers.

## 2. Materials and Methods

### 2.1. Study Design and Ethics

This was a parallel-arm pilot randomized controlled trial (RCT) addressing the feasibility and preliminary efficacy of the Move, Eat, and Sleep m-health intervention in shift-workers in a four-week pilot trial. Following eligibility screening, provision of informed consent, and baseline assessments, participants were randomly allocated using permuted block randomization to either an intervention group or a wait-list group (access to intervention after final assessment at four weeks). The trial was registered with the Australian New Zealand Clinical Trials Registry (ACTRN12618001785291) and adhered to CONSORT guidelines [30] This study was approved by the University of Newcastle Human Research Ethics Committee (H-2017-0039). Participants were given a $50 gift-card upon completion of the final assessment.

### 2.2. Intervention Development

The intervention in this feasibility study was adapted from interventions that specifically targeted activity and sleep, with or without diet behaviors, but intentionally excluded shift-workers, (“Synergy” ACTRN12617000376347, “Refresh” ACTRN12617000680369, and “Move, Eat, and Sleep” ACTRN12617000735358) [29,31,32,33]. In these previous trials, shift-workers were specifically excluded because of their unique requirements, discussed in the introduction of this paper. The development of the intervention content was guided by using specific behavior change techniques (e.g., education, goal-setting, self-monitoring, feedback on behavior) to operationalize constructs from social cognitive and self-regulatory theories (for further information, please see Table S2, Supplementary Materials) [33,34,35,36,37]. Due to the cost of modifying the app and the need for specific feedback from the target audience on key features that need to be added or improved to increase its utility for shift-workers, the app was used in its original form in the current study. However, the handbook distributed to the intervention group, while based on that used in the original “Move, Eat, and Sleep” intervention, was tailored to the shift-worker population. Examples of the tailored content included the use of strategies to better manage switching between shift and non-shift days, such as minimizing sleep debt before starting a string of night shifts. The weight-loss chapter was excluded as the current intervention was not focused on weight loss but rather diet quality and meal planning.

### 2.3. Participants and Recruitment

The study recruited shift-workers aged 18–65 years from all over Australia using social media advertising (Facebook). Eligibility criteria included being insufficiently active (<150 min per week of moderate to vigorous activity (MVPA)) as assessed via the Active Australia Questionnaire [38], reporting poor diet quality (score ≤ 6 out of 10 for a short screening questionnaire; Table S1, Supplementary Materials), and/or rating their sleep as poor- or very-poor-quality sleep using a single-item from the Pittsburgh Sleep Quality Index (PSQI) [39], plus having access to an internet-enabled iOS or Android smart phone and residing in Australia. Exclusion criteria included having a condition that makes it unsafe to change physical activity, diet, or sleep, and reporting a body mass index (BMI) <18.5 or ≥40 kg/m^2^. Eligible participants completed an online baseline questionnaire and were randomized using sequentially numbered opaque envelopes into the intervention or wait-list group. Those allocated to the intervention group were emailed the details to download and log in to the intervention platform. A link to the follow-up questionnaire was distributed at four weeks, and, during weeks five and six, interviews with intervention-group participants were conducted and recorded via Zoom (Zoom Video Communications, San Jose, CA, USA) by the study coordinator (S.O.) to collect qualitative feedback for further improvements of the app. The app prompted participants daily if they did not enter data.

### 2.4. Wait-List Group

Participants were asked to maintain usual lifestyle habits in the four weeks between assessments and gained access to the intervention after study completion.

### 2.5. Intervention Group

The intervention components are described below. 

#### 2.5.1. Balanced App 

The intervention [40] was delivered via an information technology (IT) platform using iOS and Android smartphone apps. A unique identifier (password + login) for each participant allowed use to be monitored. Participants set goals for and self-monitored physical activity (i.e., minutes of MVPA, sessions of resistance training), diet quality (i.e., number of serves of core foods, soft drink/fast food/alcohol-free days), and sleep (i.e., duration, quality, hygiene, and variability (time to bed and awake)) and updated these goals in line with their progress. Participants were encouraged to log daily by an automated push-notification from the app. Personalized feedback on progress toward goals was provided using graphs within the app on a daily, weekly, and monthly basis. The Balanced app uses a traffic light feature on the app’s home screen (dashboard) to provide participants with dynamic feedback on their performance relative to their goal. A green light indicates a participant is meeting, exceeding, or close to their goal. an orange light indicates they are progressing somewhat toward their goal. and a red light indicates they are markedly below their goal.

#### 2.5.2. Weekly Summary Reports

Detailed feedback reports (e.g., most active days, number of days where goals were achieved) were generated based on data downloaded from the IT platform, using custom STATA do-files, and emailed to participants.

#### 2.5.3. The Shift-Worker Move, Eat, and Sleep Handbook

The handbook explained the physical, mental, and social benefits of improving health behaviors and included tools for action planning. The main topics with sub-chapters were (a) goal-setting for physical activity (finding an enjoyable movement and action plan for physical activity, including a weekly activity planning template); (b) goal-setting for healthy eating (optimizing food variety, hunger/fullness awareness, meal planning, and an action planning template for eating habits and food variety, including a hunger/fullness diary and a meal planning and grocery shopping template); (c) goal-setting for sleep (action planning template for sleep and sleep hygiene behaviors); (d) mindfulness practice and stress reduction (mindful eating and movement, mindfulness meditation for sleep, stress management techniques). The handbook was delivered to participants as a pdf document via email.

#### 2.5.4. Weekly SMS

Weekly messages were delivered via short message service (SMS) were scheduled with facts and tips for improving physical activity, diet quality, and sleep.

### 2.6. Data Collection

Participants were assessed at baseline and at four weeks. Questionnaires were completed via an online portal and qualitative interviews via Zoom. The interviews were recorded after receiving participant consent to allow transcription.

### 2.7. Feasibility Measures

Feasibility of research procedures was assessed via weekly recruitment rates and cost per allocated participant. Potential participants who met eligibility criteria, consenting participants who completed baseline assessment, participants who commenced their randomly allocated treatment, and participants who completed all follow-up assessments were tracked. The capacity and resources needed to complete all study processes were assessed via time taken to complete study processes.

### 2.8. Implementation Outcomes

The implementation outcomes were assessed as recommended by Proctor et al. [41]. User engagement with the intervention platform was assessed by actual participant-entered data into the app. The number of participants engaging with the app during the four weeks of the intervention and the number of self-monitoring days logged were extracted. Participants rated the intervention’s usefulness via the online survey in terms of “increasing confidence”, “overcoming barriers”, “planning”, and “staying motivated” for change in physical activity, diet, and sleep on a five-point scale (1 = not useful at all), and the proportion who rated the intervention “slightly useful” or better was reported. Participants also rated the usefulness of intervention components on a five-point scale (3 = neither agree nor disagree) and the proportion who “agreed” or “strongly agreed” that it was useful was reported. The app was rated using the System Usability Scale which is a 10-item attitude Likert scale giving a subjective assessment of usability (effectiveness, efficiency, and satisfaction) with a total score ranging from 0 to 100 [42]. Semi-structured qualitative interviews of the intervention group were conducted by the study coordinator (S.O.) and took around 30 min via Zoom. Participants were asked a series of questions in relation to motivations for enrolling, perceived acceptability, and appropriateness of the individual components of the program in terms of which elements they found beneficial and which elements need improvement, and lastly if the program met their expectations. Prompts included the physical activity, diet, or sleep components in the app and handbook, the app itself, the handbook itself, the weekly SMSs and progress reports, and the stress management information.

### 2.9. Estimation of Treatment Effect

Physical activity was self-reported using the Active Australia Questionnaire and two separate items asking how many sessions of muscle-strengthening activities they participated in last week, and the average duration of these sessions [33]. Diet quality was assessed using a 70-item food frequency questionnaire, which was then used to produce the Australian Recommended Food Score (ARFS) [43]. The ARFS focuses on dietary variety within food groups (i.e., fruits, vegetables, grains, meats and alternatives, dairy) and aligns with the Australian Dietary Guidelines [44]. Sleep quality was measured using the Pittsburgh Sleep Quality Index (PSQI), a valid and reliable instrument widely used to assess change in sleep quality in public health interventions [45]. The PSQI assesses the important restorative effects of sleep that can only be assessed by self-report, and provides measures of subjective sleep quality, sleep onset latency, sleep duration, sleep efficiency, sleep disturbances, use of sleeping medication, and daytime dysfunction, which are components of sleep health [45]. A global score is calculated and a score >5 is indicative of poor sleep [45].

Additional measures included were take-away frequency (i.e., breakfast, lunch, dinner, and snacks) and frequency of consuming discretionary foods (e.g., chocolate, lollies, pies, pastries, cakes, fried potato, fast food, and soft drink). Eating competence was assessed using the Satter Eating Competence (“ecSatter”) inventory 2.0 [46]. The ecSatter inventory is designed to empirically assess constructs of the Satter eating competence model which conceptualizes functional eating attitudes and behavior and gives an overall eating competence score, where ≥32 indicates eating competence [46]. Risk of sleep apnea was assessed using the Berlin questionnaire [47]. Sleep hygiene was assessed using the Sleep Hygiene Index [48]. Sociodemographic characteristics (i.e., age, gender, ethnicity, education, marital status, postcode, employment status), shift-work characteristics, work hours, height and weight to calculate body mass index (BMI, kg/m^2^), smoking, alcohol consumption, presence of chronic diseases, and medication use were assessed at baseline only.

### 2.10. Sample Size

CONSORT guidelines state that sample size requirements for pilot studies are based on those necessary to assess the practicalities of the protocols, and not the requirements necessary for assessing intervention efficacy. As such, it was anticipated that 20 participants per group, or a total of 40 participants would be adequate.

### 2.11. Qualitative Analysis

Interviews were transcribed verbatim and summarized under the categories of motivations for enrolling, perceived acceptability and appropriateness of the individual components of the program in terms of which elements they found beneficial and which elements need improvement, and if the program met expectations. Responses were then coded and categorized into themes within each of these categories by the study-coordinator.

### 2.12. Statistical Analysis

Descriptive statistics were reported as counts (%) and means (SD). Preliminary intervention effects were tested using mixed model analysis, which is consistent with the intention-to-treat (ITT) principle. Differences of means and 95% confidence intervals (CIs) were determined. Separate models were estimated for each outcome, and included fixed effects for time (categorical: baseline and four weeks), treatment group (intervention and wait-list), and an interaction term for time-by-treatment group and a random intercept for individuals to account for the repeated measures. The *p*-value associated with the treatment term was used to determine the statistical significance of any differences between treatment groups (*p* < 0.05). Effect sizes were calculated using the following equation: Cohen’s *d* = (mean_1 change score_ − mean_2 change score_)/SD _pooled, change scores_ [49].

## 3. Results

Sample characteristics (*n* = 40) at baseline are presented in Table 1 and health behaviors are presented in Table 2. The majority of participants worked as a shift-worker for ≥7 years, did not live away from home for work, and worked rotating shifts of 9.9 (2.0) hours per shift. All participants reported doing ≥300 min of MVPA per week, and 45% met physical activity guidelines (both ≥150 min of MVPA and ≥2 sessions of muscle-strengthening activity per week). Nearly 55% of participants fell in the lowest diet quality category “needs work”, and only 6% scored “excellent”. Eating competence was low in 73% of the participants and average weekly take-away frequency was 4.9 (2.8) times per week. Mean PSQI score was 7.7 points, with 78% in the “poor quality” category. Approximately 38% of participants (*n* = 15) were at high risk of sleep apnea, and 35% (*n* = 14) reported falling asleep while driving a vehicle.

### 3.1. Feasibility of Research Procedures

#### 3.1.1. Recruitment

**Figure 1:**
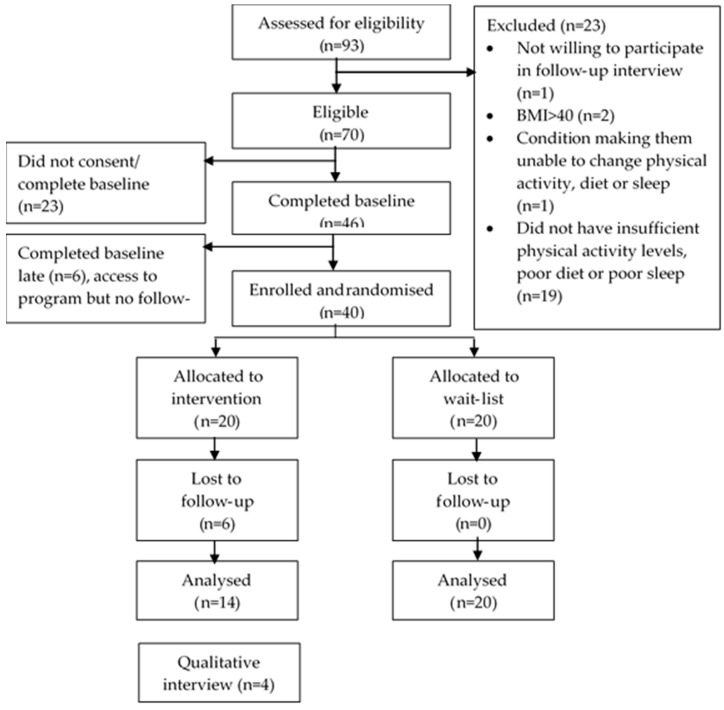
CONSORT flow chart describing the progress of participants through the trial. Flow of participants through the four-week shift-worker Move, Eat, and Sleep pilot study.

Three Facebook adverts were utilized, each running for six hours on three separate evenings within one week in October 2018. The first ad targeted women only by instructing Facebook to show it only to females, while the next two targeted only men (Figure S1, Supplementary Materials). The ads cost AUD$400 in total, equating to a cost of $8.70 per randomized participant. Of the 93 individuals who completed the eligibility survey, 70 were deemed eligible, and 46 completed the baseline survey, of whom 40 were enrolled and randomized (Figure 1). At the four-week follow-up, overall participant retention was 85% (34/40). Six participants were lost to follow-up and could not be reached to obtain a reason.

#### 3.1.2. Reminders

In total, two emails and two SMS reminders were sent to complete baseline and follow-up surveys over the course of one week.

#### 3.1.3. Capacity and Resources 

The recruitment and randomization phase and the follow-up and interview phase required one staff member full-time for a week, while the four-week intervention phase required one staff member half a day per week.

### 3.2. Implementation Outcomes

#### 3.2.1. User Engagement and Retention

Data from the app platform showed that 70% (14/20) used the Balanced app at least once. Ten percent (2/20) ceased use after the first week, 5% (1/20) ceased use after week three, 10% (2/20) commenced use late (weeks two and three), and, in week four, 55% (11/20) were still using the app. The mean number of days logged for those who used the app at all (70%, 14/20) was 11.6 (10.6) days out of 28. The majority of responding participants (92.9%, 13/14) reported reading some or all of the handbook.

#### 3.2.2. Acceptability of Overall Intervention and Its Components

The proportion of participants who scored the intervention as “slightly useful” or above in terms of “increasing confidence”, “overcoming barriers”, “planning”, and “staying motivated” to participate in physical activity, healthy food habits, and sleep habits ranged from 46% (6/13) to 85% (11/13) (Table 3). Overall 58.3% and 69.2% of participants agreed or strongly agreed that the Balanced app and handbook was useful, respectively (Table 3). Between 25% and 69% (4/13 to 9/13) agreed that the individual sections of the app and handbook were useful. The System Usability Scale score for the app was 62.7 (12.7) points out of 100, indicating “below average” usefulness (68 point cut-off is equal to “average”).

#### 3.2.3. Qualitative Interviews

Only 20% of participants (4/20) in the intervention group completed the qualitative interview and 35% (7/20) entered free text in the survey, with an overlap between these participants. Four main themes emerged. Firstly, there was frustration if behaviors could not be recorded as accurately as they wished, for example, “*track actual hours slept when having multiple sleeps*”, “*actual pieces of fruit and vegetables eaten*”, and “*log walking, moderate, and vigorous intensity activity separately*”. Participants reported losing motivation due to inability to track behaviors “accurately” by their own definition. There was accountability by checking tracking on app regularly. Participants reported the app helped them “*stay honest*”, “*check in at the end of the day*”, “*be accountable*”, and “*be more mindful*”, and acted as a “*reminder to do better tomorrow*” in terms of their physical activity, diet, and sleep behaviors. They liked the traffic-light tiles, and one participant stated not wanting to get a red tile: “*even on the day shifts I have to wake up at 3:30 in the morning. If I don’t go to bed before sort of… if I go to bed after about 9:30, the app doesn’t like it. Neither do I just quietly. But it just… I suppose it makes me realize, oh, its day shift tomorrow, I’ve got to make sure I get to bed on time*”. Thirdly, the handbook was too long: “*I think it was probably just too… I sort of looked at it and went, that’s a lot of reading. I don’t normally read that much.*” It was suggested that it would be easier to read if split into smaller sections either accessed through the app or sent via email at intervals. One participant suggested a smaller amount of information could be delivered with a daily challenge, similar to a “quit smoking” app she successfully used in the past. Lastly, additional information or features such as linking to resources such as recipes, basic strength training guides, and mindfulness apps, and syncing to other apps to avoid double-tracking (activity trackers/step-counters) were suggested.

### 3.3. Estimation of Treatment Effect

A summary of intervention effects can be found in Table 4. No significant between-group differences were found for time spent in MVPA (307 min/day; 95% CI = 638 to 24; *p* = 0.069) or sleep quality (0.24 points; 95% CI = 0.7 to 2.2; *p* = 0.806). A significant improvement in diet quality in the intervention group compared to the wait-list group was found, with a between-group difference of 4.5 points (95% CI = 0.1 to 8.9; *p* = 0.047). For the secondary measures, there were no significant between-group differences for sleep hygiene, eating competence, and frequency of fast food or discretionary food consumption. The proportion of participants meeting physical activity guidelines (≥150 min of MVPA plus ≥2 sessions of muscle-strengthening activity per week) in the intervention group increased by 14.3% due to an increase (2/14) in participants meeting the muscle-strengthening guideline, while there was no change for the wait-list group.

## 4. Discussion

This pilot RCT tested the feasibility of the shift-worker Move, Eat, and Sleep intervention in Australian shift-workers aged 18–65 years, who reported either insufficient moderate-to-vigorous physical activity, poor-quality sleep, or a poor-quality diet. The research procedures (recruitment, randomization, data collection and retention) were feasible, with 40 eligible shift-workers recruited in a week and low attrition with 85% completing the follow-up questionnaire. User engagement was acceptable compared to other app-based interventions [50], with 70% accessing the app at least once and 55% still using the app at four-weeks follow-up. The proportion accessing the app at least once was comparable to that of other app-based interventions [50], but as it is well known that engagement tapers off with time in technology-based interventions, studies with longer follow-up time often report lower engagement rates at the end of the study [51]. When used in a non-shift-worker population, the app scored better on the system usability score, with 70.8 (19.7) points compared to the shift-worker population with 62.7 (12.7) points [33]. The non-shift-workers also used the app for longer, with an average of 37.0 days of data logged compared to 11.6 days in the shift-worker population; however, as a proportion of study length, the logging events were similar (44% of 84 days vs. 41% of 28 days, respectively) [33]. As it was shown that engagement with the intervention (i.e., app) significantly predicts behavior change, understanding why participants disengage is critical to improving the app for future use [52].

The quantitative and qualitative feedback on implementation outcomes indicated a need for modifications to both the app and handbook to better suit the shift-worker population. Key modifications for future versions of interventions for shift-workers are suggested below.

Ability to track multiple sleep periods within one day. Date and a.m./p.m. or 24-h time when adding sleep periods could be included to lessen confusion when entering.

More detailed tracking in the app to be able to see progress better (i.e., different activity levels/types, number of serves of fruit and vegetables compared to just ticking, e.g., if they had five servings of vegetables or not, and a tally of total hours of sleep when having multiple sleeps). This relates back to building self-efficacy and goal-setting (Table S2, Supplementary Materials), with participants indicating they would like to measure more incremental progress toward their goal.

A different format for the handbook to improve acceptability and accessibility, yet still allowing scalability. Delivering the information incrementally via regular emails, via a website with interactive action planning tools, as integrated chapters in the app, or as a combination of these may be feasible options. Making the information more accessible for those with lower levels of literacy may also be required by using simpler language, presenting information in audio-visual formats (i.e., short videos in the app), tailoring content to individual needs (i.e., based on goals), and other forms of interactivity [53].

Introduce interactive features to encourage engagement and “checking in”, for example challenges or automated messages with update on progress. This relates back to increasing self-efficacy by using challenges, praise, and rewards as a type of relapse prevention.

These recommendations are based on the findings that, while 76.9% found the overall intervention slightly or moderately useful in motivating them to participate in more physical activity, sleep, and healthier eating habits, only half or less (41.7% to 50.0%) of the participants found the “move”, “eat”, and “sleep” sections in the app useful. Those who completed the qualitative interviews noted a lack of motivation to continue using the app because it did not allow them to log what they would like to log, i.e., it was not tailored to their needs. Furthermore, the information in the handbook tended to rank higher (61.5–69.2%) than the planning tools and action plans included (30.8–61.5%), perhaps because they were provided and emailed in a pdf document and not as print material. From the qualitative interviews, it was clear that the primary barrier for using the handbook was its length and, despite having intentions of returning to read more at a later time, not doing so.

These recommended changes broadly fit into the categories of “tailoring to individual needs” and “combining digital behavior change interventions (DBCIs) with human support”, which were issues identified in an expert consensus paper regarding promoting effective engagement with DBCIs [53]. To examine the program in a format that would allow scalability, the current intervention did not include any human contact apart from emails conveying information on eligibility, group allocation, and automated progress reports. This may have limited uptake of the intervention and engagement with the app. Although few studies directly contrasted different levels of support [53], some evidence suggested that multi-component as opposed to stand-alone app interventions may be more effective [52]. Gamification by adding challenges within the app was suggested by one of the participants interviewed and may increase engagement. Social features within the app or via a third-party medium (Facebook/Snapchat/WhatsApp group, website with chat rooms, etc.) are also a possibility, but would require greater resources due to the need for a facilitator and modifier within those spaces. Further research is needed to determine which features of apps increase engagement [52,54,55].

The current study also aimed to estimate the treatment effect of the shift-worker Move, Eat, and Sleep intervention on primary outcomes (minutes of moderate-to-vigorous physical activity, diet quality, and sleep quality) and secondary outcomes (percentage meeting physical activity guidelines, frequency of take-away purchases, consumption of discretionary foods, Sleep Hygiene Index). Findings indicated a positive effect on diet quality, while no significant effect was observed for other measures. At the start of the intervention, the entire sample (*n* = 40) reported exceeding 300 min of MVPA a week, which is twice the minimum minutes of MVPA recommended [56] and far higher than Australian population average [57]. At follow-up, the minutes of MVPA in the intervention group was significantly lower in the intervention group compared to the wait-list group (−307 min/day; 95% CI = −638 to 24; *p* = 0.069, *d* = 0.67). This may be because the intervention group was more aware of the activity they were doing and recorded it more accurately, or the validity of the assessment method. Furthermore, the high levels of activity at baseline likely limited any potential for improvement. The use of activity monitors (pedometers or accelerometers) to objectively record baseline and follow-up activity may improve the accuracy of results. Both the wait-list and intervention groups reported a slight improvement in sleep quality, with a non-significant group difference, but the intervention time frame may not have been sufficient to demonstrate changes in sleep. While a meta-analysis of interventions in individuals without a sleep disorder with an average of five weeks duration (range: 2–10 weeks) was found to have a significantly positive effect on sleep quality [58], in individuals with insomnia, longer treatment duration was associated with larger effect sizes (range: 4–48 weeks) [59]. It is possible that the inherent changing schedule of shift-workers necessitates a longer intervention time to see the benefits of implementing improved sleep hygiene practices.

### Limitations

The use of Facebook to recruit may have introduced a recruitment bias, and the use of self-report introduces reporting bias. The fact that all participants reported 300+ min of MVPA likely highlights the latter point. The sample may not be representative of the shift-worker population as a whole, as only 10% (*n* = 4) lived away from home while working, and the sample was also primarily Caucasian. The small sample size may have influenced the magnitude and variability of the effect sizes described. Also, the current intervention did not consider workplace level factors or environmental characteristics (i.e., access to fresh food, fruit, and vegetables) which may be important to consider in future interventions [60]. The difficulties in completing the qualitative interviews at follow-up limited the feedback available for modifying the app. Finally, as this was a pilot study, it was not powered to detect changes in health behaviors.

## 5. Conclusions

The intervention demonstrated feasibility in shift-workers in terms of recruitment and study resources, and provides guidance on modifications required to tailor the intervention components to shift-worker needs to increase engagement.

## Figures and Tables

**Table 1:** Sociodemographic and health descriptors of shift-worker Move, Eat, and Sleep participants (*n* = 40).

Variable and Measurement Scale/Category		Intervention	Wait-List	Total
		Mean (SD)		
Age	Years	34.9 (9.2)	36.6 (10.1)	35.7 (9.5)
Education	Years	16.7 (2.5)	15.4 (3.2)	16.0 (2.9)
Body mass index	Weight, kg/height, m^2^	26.3 (4.3)	29.9 (5.4)	28.1 (5.2)
Shifts last week	Count	4.1 (1.2)	4.2 (1.6)	4.2 (1.4)
Length of shifts	Hours	10.0 (2.1)	9.9 (2.0)	9.9 (2.0)
Depression Anxiety and Stress Scale-21	Depression (0–42) ^1^	9.6 (9.3)	8.0 (7.7)	8.8 (8.5)
	Anxiety (0–42) ^2^	4.7 (4.6)	5.5 (5.8)	5.1 (5.2)
	Stress (0–42) ^3^	12.0 (8.0)	13.8 (7.3)	12.9 (7.6)
		Count (%)		
Sex	Female	12 (60)	9 (45)	21 (52.5)
	Male	8 (40)	11 (55)	19 (47.5)
Ethnic background	Caucasian	18 (90)	18 (90	36 (90)
	Aboriginal, Torres Strait, or Pacific Islander	-	1 (5)	1 (2.5)
	Asian	1 (5)	1 (5)	2 (5)
	Middle Eastern	1 (5)	-	1 (2.5)
Marital status	Partnered	15 (75)	14 (70)	29 (72.5)
	Not partnered	5 (25)	6 (30)	11 (27.5)
Individual income per annum (gross)	≤AUD$70 k/annum	8 (30)	9 (45)	16 (40)
	>AUD$70 k/annum	12 (70)	10 (50)	13 (57.5)
	Do not know	-	1 (5)	1 (2.5)
Employment status	Full time	12 (60)	14 (70)	26 (65)
	Part time	5 (25)	5 (25)	11 (27.5)
	Causal	1 (5)	-	1 (2.5)
	Other	1 (5)	1 (5)	2 (5)
Live away from home for work	Yes	1 (5)	3 (15)	4 (10)
	No	19 (95)	17 (85)	36 (90)
Work pattern	Mainly night	2 (10)	-	2 (5)
	Rotating	13 (65)	14 (70)	27 (67.5)
	Some days, afternoons, and nights	4 (20)	5 (25)	9 (22.5)
	Some days and afternoons/mainly afternoons	1 (5)	1 (5)	2 (5)
Length of time as shift-worker	≤2 years	4 (20)	2 (10)	6 (20)
	3–6 years	4 (20)	7 (35)	11 (27.5)
	≥7 years	10 (50)	11 (45)	21 (52.5)
Hours worked last week (total)	≤30 h	4 (20)	3 (15)	7 (17.5)
	31 to 50 h	13 (65)	15 (75)	28 (70)
	>50 h	3 (15)	2 (10)	5 (12.5)
Berlin questionnaire ^4^	High risk of sleep apnea	6 (30)	9 (45)	15 (37.5)
	Low risk sleep apnea	14 (70)	11 (55)	25 (62.5)
Fallen asleep while driving	Yes, 1–2 times per month	3 (15)	2 (10)	5 (12.5)
	Yes, but “nearly never”	5 (25)	4 (20)	9 (22.5)
	Not fallen asleep	12 (60)	14 (70)	26 (65)
Chronic disease	Yes	7 (35)	10 (50)	17 (42.5)
	No	13 (65)	10 (50)	23 (57.5)
Self-rated health	Poor to fair	8 (40)	7 (35)	15 (37.5)
	Very good to excellent	12 (60)	13 (65)	25 (62.5)

Depression Anxiety and Stress Scale-21: ^1^ Depression: 0–9 = normal, 10–13 points = mild symptoms; ^2^ anxiety: 0–7 = normal, 8–9 points = mild symptoms; ^3^ stress: 0–15 = normal, 15–18 points = mild symptoms. ^4^ Berlin questionnaire: ≥2 points = high risk of sleep apnea.

**Table 2:** Health behavior indicators for shift-worker Move, Eat, and Sleep participants at baseline (*n* = 40).

Variable and Measurement Scale/Category		Intervention (*n* = 20)	Wait-List (*n* = 20)	Total (*n* = 40)
		Mean (SD)		
Average moderate to vigorous physical activity (MVPA) ^1^	Average minutes	1462 (300)	1198 (450)	1330 (401)
Australian Recommended Food Score (ARFS)	Average score	31.2 (7.0)	32.7 (7.2)	31.9 (7.0)
Take-away (breakfast, lunch, dinner, and/or snacks)	Average times per week	4.2 (2.7)	5.7 (2.7)	4.9 (2.8)
Discretionary foods ^2^	Average score per item ^3^	2.5 (1.1)	2.9 (1.0)	2.7 (1.0)
	Total score ^4^	12.6 (5.3)	14.3 (4.9)	13.4 (5.1)
Sitting time (hours) ^5^	Average per day	6.4 (3.7)	7.7 (4.6)	7.1 (4.2)
Pittsburgh Sleep Quality Index score	Total score	7.5 (4.3)	7.9 (3.3)	7.7 (3.8)
Sleep Hygiene Index ^6^	Total score (0–60)	39.5 (6.4)	36.4 (5.9)	37.9 (6.3)
Eating competence score ^8^	Total score (0–48)	27.4 (7.3)	25.1 (8.8)	26.2 (8.1)
		*n* (%)		
Meet physical activity recommendations ^7^	No	11 (55)	11 (55)	22 (55)
	Yes	9 (45)	9(45)	18 (45)
Sitting time category	>8 h per day	8 (40)	9 (45)	17 (42.5)
	≤8 h per day	12 (60)	11 (55)	23 (57.5)
Eating competence category ^9^	Low eating competence	29 (72.5)	25 (73.5)	54 (73.0)
	Eating competent	11 (27.5)	9 (26.5)	20 (27.0)
Diet quality category (ARFS categories)	Needs work (0–33)	11 (57.9)	9 (50.0)	20 (54.5)
	Getting there (34–38)	5 (26.3)	6 (33.3)	11 (29.7)
	Excellent (39–46)	3 (16.7)	3 (15.8)	6 (16.2)
	Outstanding (47+)	-	-	-
Pittsburgh Sleep Quality Index category ^10^	Poor sleep quality	14 (70)	17 (85)	31 (77.5)
	Good sleep quality	6 (30)	3 (15)	9 (22.5)

^1^ Average MVPA = walking minutes + moderate activity minutes + (2 × vigorous activity minutes), max = 1680 min. ^2^ Discretionary foods, five items: confectionary (chocolate, lollies)/biscuits, cakes, pies, cake-type desserts, pastries/fried potato, French fries, hot chips, wedges, hash brown/fast food (e.g., pizza, burgers, Chinese, Kentucky Fried Chicken/fried chicken, fried fish)/soft drink, energy drinks, sports drinks or flavored milks. ^3^ Scored: 0 = never, 1 = less than once a month, 2 = 1–3 times per month, 3 = 1/week, 4 = 2–4/week, 5 = 5–6/week, 6 = 1/day, 7 = 2+/day. ^4^ Summed score ranges 0 to 35, max score equals eating/drinking each of these 2+ times per day. ^5^ Average sitting time work days and non-work days. ^6^ Sleep Hygiene Index, score ranges 0–60, higher score indicates poorer sleep hygiene. ^7^ Meeting physical activity recommendations: ≥150 min of MVPA plus two muscle-strengthening sessions per week. ^8^ Ellyn Satter Eating Competence (ecSatter2.0). ^9^ ecSatter2.0 score ≥32 indicates eating competence. ^10^ Pittsburgh Sleep Quality Index score >5 indicates poor-quality sleep.

**Table 3:** Usefulness of shift-worker Move, Eat, and Sleep intervention overall (*n* = 13, *n* = 1 did not access any of program).

Physical Activity: How Useful Was Shift-Worker Move, Eat, and Sleep in Helping You (*n* = 13)	Slightly to Moderately Useful (*n* (%))
Increase your confidence for engaging in regular physical activity over the past 4 weeks?	10 (76.9)
Overcome barriers to participating in physical activity over the past 4 weeks?	10 (76.9)
To plan for physical activity over the past 4 weeks?	9 (69.2)
To stay motivated to participate in physical activity over the past 4 weeks?	10 (76.9)
Food: How useful was shift-worker Move, Eat, and Sleep in helping you (*n* = 13)	
Increase your confidence for engaging in healthy food habits over the past 4 weeks?	11 (84.6)
Overcome barriers to engaging in healthy food habits over the past 4 weeks?	9 (69.2)
To plan for healthy food habits over the past 4 weeks?	9 (69.2)
To stay motivated to engage in healthy food habits over the past 4 weeks?	10 (76.9)
Sleep: How useful was shift-worker Move, Eat, and Sleep in helping you (*n* = 13)	
Feel confident in prioritizing my sleep needs in the past 4 weeks?	6 (46.2)
Overcome barriers to healthy sleep habits in the past 4 weeks?	10 (76.9)
Plan for healthy sleep habits over the past 4 weeks?	11 (84.6)
Stay motivated to engage in healthy sleep habits over the past 4 weeks?	10 (76.9)
Handbook items (*n* = 13 as *n* = 1 did not read any of the handbook)	Agree to Strongly Agree
The handbook was useful	9 (69.2)
The physical activity information was useful	9 (69.2)
The physical activity action plan was useful	6 (46.2)
The information on food variety was useful	9 (69.2)
The tools for increasing food variety were useful	8 (61.5)
The hunger/fullness awareness was useful	7 (53.9)
The hunger/fullness diary was useful	6 (46.2)
The information on food planning was useful	4 (30.8)
The meal planning tool was useful	4 (30.8)
The action plan for eating habits and food variety tool was useful	5 (38.5)
The sleep information was useful	8 (61.5)
The action plan for sleep was useful	5 (38.5)
The relaxation and stress management section was useful	7 (58.3)
The goal-setting information was useful	7 (53.9)
App items (*n* = 12 as *n* = 2 did not download the app)	
The Balanced App was useful	7 (58.3)
Physical activity section of app was useful	6 (50.0)
It was easy to personalize my activity goals	8 (66.7)
It was easy to track my physical activity	7 (58.3)
Food section of app was useful	6 (50.0)
It was easy to personalize my food goals	7 (58.3)
It was easy to track my food goals	7 (58.3)
Sleep section of app was useful	5 (41.7)
It was easy to personalize my sleep goals	7 (58.3)
It was easy to track my sleep goals	7 (58.3)
Progress graph in app useful	3 (25.0)
Resources in app were useful	4 (33.3)
Other components (*n* = 13)	
The weekly reports were useful	7 (53.8)
The weekly SMSs were useful	9 (69.2)
The multiple parts of the intervention were not overwhelming	10 (76.9)
The program was able to be personalized enough to match my goals	9 (69.2)
There was enough contact with the study coordinators	8 (61.5)
System Usability Scale	
System Usability Scale score (0–100 points) (*n* = 13)	62.7 (12.7)

**Table 4:** Estimation of intervention effect, mixed model analysis (intention-to-treat).

	Baseline		Follow-Up		Mean Change from Baseline (95% CI)		Mean Difference between Groups (95% CI)	Group × Time *p*-Value	Effect Size (Cohen’s *d*)
Outcomes	Wait-List (*n* = 20)	Intervention (*n* = 20)	Wait-List (*n* = 20)	Intervention (*n* = 14)	Wait-List	Intervention			
Moderate to vigorous physical activity minutes ^1^	1120 (390 to 1680)	1462 (660 to 1680)	1273 (360 to 1680)	1248 (330 to 1680)	75 (−144 to 294)	−232 (−480 to 16)	−307 (−638 to 24)	0.069	0.67
Australian Recommended Food Score, total score	33.2 (29.7 to 36.6)	31.0 (27.6 to 34.5)	33.2 (29.7 to 36.6)	35.6 (31.6 to 39.5)	0.01 (−2.8 to 2.8)	4.5 (1.1 to 7.9)	4.5 (0.1 to 8.9)	0.047	0.76
Pittsburgh Sleep Quality Index, total score	7.9 (3 to 15)	7.5 (2 to 18)	7.1 (3 to 13)	7 (2 to 13)	−0.8 (−2.1 to 0.5)	−1.0 (−2.5 to 0.4)	0.2 (−0.7 to 2.2)	0.806	0.19
Proportion meeting physical activity guidelines ^2^	9 (45.0)	10 (50.0)	9 (45.0)	9 (64.3)	No change	+14.3% ^3^	-	-	-
Eating competence	25.1 (8 to 42)	27.1 (14 to 43)	27.4 (10 to 41)	31.1 (18 to 42) ^4^	2.1 (−2.3 to 6.4)	3.7 (−0.3 to −7.2)	−1.5 (−8.1 to 5.1)	0.650	-
Frequency of fast food	5.7 (0 to 10)	4.2 (0 to 10)	6.2 (1 to 10)	3.6 (0 to 10)	0.5 (−0.6 to 1.6)	−0.4 (−1.8 to 0.9)	0.9 (−0.8 to 2.7)	0.298	-
Frequency of discretionary foods	2.9 (1 to 4.8)	2.5 (0.4 to 4.6)	2.9 (0.8 to 4.8)	2.2 (0.4 to 3.6)	0.0 (−0.32 to 0.31)	−0.3 (−0.7 to 0.1)	−0.3 (−0.8 to 0.2)	0.196	-
Sleep Hygiene Index	36.4 (5.9 (21 to 45)	39.5 (29 to 51)	37.6 (20 to 54)	39.9 (29 to 53)	1.6 (−0.9 to 4.0)	0.6 (−2.2 to 3.5)	0.9 (−2.8 to 4.7)	0.623	-

^1^ Active Australia Questionnaire. ^2^ Participating in ≥150 min MVPA plus two muscle-strengthening sessions per week. ^3^ 14.3% (*n* = 2) of remaining (*n* = 14) with follow-up data changed from not meeting to meeting both PA guidelines. ^4^ *n* = 1 excluded as answered 0 to all questions. CI = confidence interval.

## Supplementary Materials

The following are available online at https://www.mdpi.com/1660-4601/16/20/3810/s1: Figure S1: Recruitment advertisements, Table S1: Screening questions for diet quality, Table S2.
