# Supplementary material for: Feasibility and Preliminary Efficacy of an m-Health Intervention Targeting Physical Activity, Diet, and Sleep Quality in Shift-Workers
Source: Int J Environ Res Public Health. 2019 Oct 10;16(20):3810. doi: 10.3390/ijerph16203810 (PMC6843501; doi:10.3390/ijerph16203810)
Supplement: Supplementary file 1 [file ijerph-16-03810-s001.pdf]

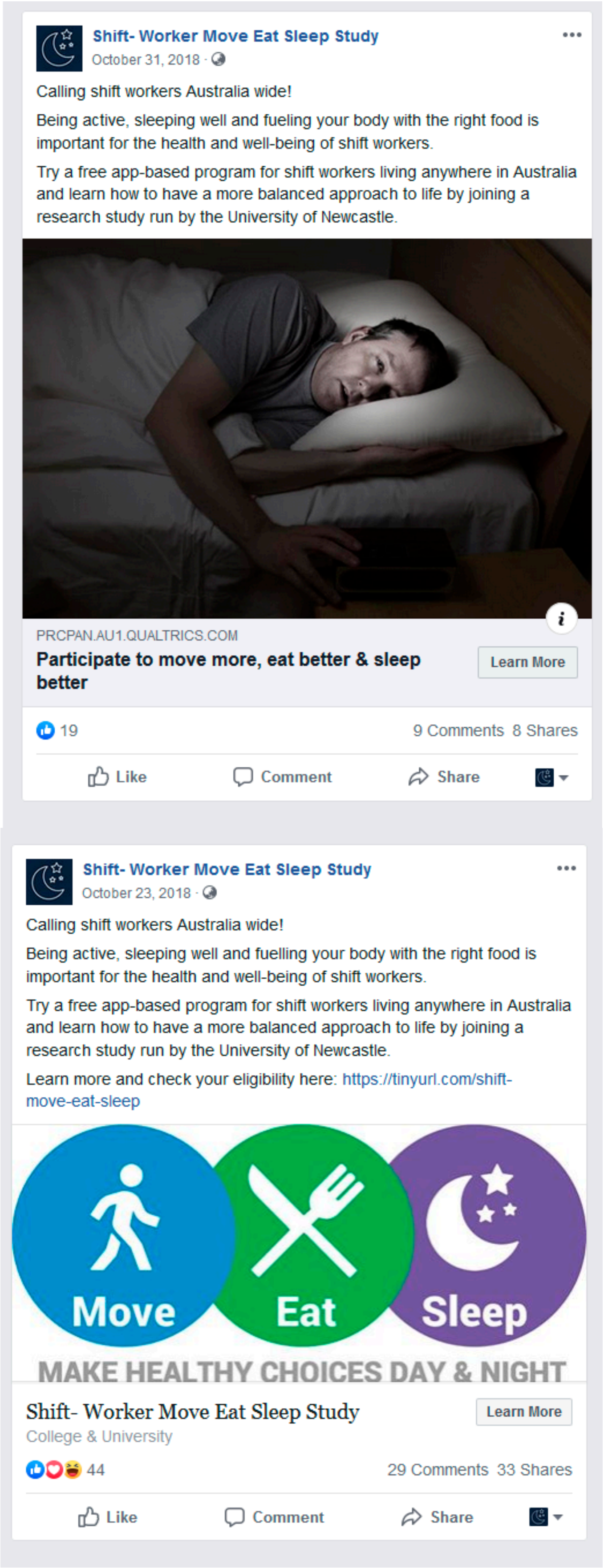

Supplemental figure 1: recruitment advertising for men (top) and women (bottom)

Supplemental Table 1: Screening questions for diet quality

Food component	Item	Minimum score (0)	Intermediate score (1)	Maximum score (2)
Vegetables	How many times a week do you eat vegetables with your meal at night? Exclude hot chips	2 or less	3-4	5 or more
	How many pieces of fruit do you usually eat each day? Include all types	Do not eat at all	1	2 or more
Grains	What type of bread do you usually eat	White<br>Don't eat bread	Other (rye, high fibre white etc.)	Brown (multigrain/ wholemeal)
Milk	What type of milk do you usually drink/use	Don't drink milk	Full cream	Skim<br>Low fat/ soy
Red meat	How many times a week do you usually eat red meat (beef or lamb), e.g. roast, chops, steak, beef stroganoff, stir fry (with or without sauce)?	5 or more times per week	Less than once a week or never<br><br>Vegetarian/vegan	1-4 times per week

Total score: a cut-off of ≤6 to be eligible for study

Table S2: Operationalisation of Psychosocial constructs and Behavior Change Techniques in the Shift-Worker Move Eat & Sleep study

Behavior Change Techniques	Intervention components
**Self-efficacy**	
- Graded tasks<br>- Self-monitoring<br>- Goal review<br>- Feedback on performance<br>- Praise/ rewards<br>- Relapse prevention/coping<br>- Barrier identification/ problem solving<br>- Stress management	**App-based log:** Participants were asked to recall and enter their activity, dietary and sleep behaviors.<br><br>**App-based progress charts:** Bar charts for daily, weekly, and 3 month progress (logged data) in relation to goal per behavior.<br><br>**App-based dashboard traffic-light:** For each of the behaviors (physical activity, diet and sleep), the dashboard produced a traffic-light color relating to whether the participant had met or exceeded goal (green), was progressing towards their goal (orange) or was markedly below their goal (red). Data could be logged and goals adjusted at any time.<br><br>**Tool sheets:** Tool sheets were included in the pdf-handbook supplied at the start of the study to promote goal setting, action planning and stress management.<br><br>**Weekly summary report (e-mail):** An overview of weekly totals and averages per behavior (if sufficient data were available and prompted participants to review goals, if needed.<br><br>**Prompts (app notifications):** Daily notifications if no data was logged in the last 24 hours.
**Perceived behavioral capability**	
- Information on where and when to be active/ meal plan and improve diet quality/ engage in sleep promoting behavior<br>- Instructions on how to be active, meal plan and improve diet quality, and engage in sleep promoting behavior	**App-based resources and pdf-handbook (educational materials):** The resources section included the current national guidelines (i.e., how much physical activity per week, how much sleep per night, dietary guidelines for serves of fruits, vegetables etc.) and brief content on the when, where, who with, and how of being active, meal planning and improving diet quality, and seeping well.<br><br>**Weekly facts (SMS):** Participants received a short text message each week with educational content on activity, diet or sleep and health to reinforce the importance of the behaviours.<br><br>**Tool sheets (pdf-handbook):** Tool sheets provided detailed information on goal setting for physical activity, meal planning and improving diet quality and sleep health, with examples of how it could be filled out.
**Outcome expectations and expectancies**	
- Information about the behavior in relation to health	**Tool sheets (pdf-handbook):** As part of the goal setting tool sheet, participants were asked to think about reasons to improve their health behaviors and what they anticipate as personal benefit of improved physical activity, diet quality and sleep (examples were provided)<br><br>**App-based resources:** Information on why activity, diet quality and sleep are important for health and well-being.<br><br>**App-based goal setting:** Participants were asked to personalize their goals, but work towards guideline recommended minima for physical activity, food group serves and sleep duration.

Goals	
- Goal setting<br>- Action planning<br>- Self-monitoring<br>- Prompt practice<br>- Time management<br>- Teach use of prompts	**App-based dashboard traffic light:** Participants were encouraged to put equal effort into improving both physical activity, diet quality and sleep.<br><br>**Tool sheets (pdf-handbook):** Participants receive goal-setting strategies and example action plans for guidance.<br><br>**Reminders:** participants were asked to set a reminder for bed time (optional) on their phone to prompt them to go to bed at a pre-planned time to encourage minimizing sleep debt.<br><br>**App-based resources:** Environmental restructuring as part of good sleep hygiene was described in the resource section and included details on how to manage he bedroom environment, especially during daytime sleep.
Socio-structural factors (social and physical environment)	
- Use of prompts<br>- Environmental restructuring<br>- Barrier identification<br>- Plan social support	**App-based resources:** Information on physical activity, diet choices and sleep in a social context and how to seek support from people in the same household (housemates, partners, and family members)<br><br>**Tool sheets:** Short examples of how to identify and manage barriers around being active, improving diet quality and getting enough sleep, and how to utilize one's social support and environment in favor of personal goals.
Note: Behavior change techniques were specified in accordance with the 40-item taxonomy of behavior change techniques by Michie et al. Psychol Health. 2011;26:1479–1498. This table was adapted from Murawski et al. Am J Prev Med. 2019;57(4):503-514.	
